# Implementation of Clinical Practice Guidelines to Prevent Cervical Cancer: Mixed Methods Study

**DOI:** 10.2196/68572

**Published:** 2025-08-15

**Authors:** Oliver C Ezechi, Folahanmi T Akinsolu, Olunike R Abodunrin, Oluwabukola M Ola, Chisom Obi-Jeff, Ishak K Lawal, George U Eleje, Joseph D Tucker, Juliet I Iwelunmor

**Affiliations:** 1 Nigerian Institute of Medical Research Lagos Nigeria; 2 Department of Public Health Lead City University Ibadan Nigeria; 3 Nanjing Medical University Nanjing China; 4 Brooks Insight Abuja Nigeria; 5 London School of Hygiene & Tropical Medicine London United Kingdom; 6 Federal Medical Centre, Birnin-Kebbi Kebbi Nigeria; 7 Nnamdi Azikiwe University Teaching Hospital Nnewi Anambra Nigeria; 8 Effective Care Research Unit, Department of Obstetrics and Gynaecology, Nnamdi Azikiwe University Awka Nigeria; 9 University of North Carolina at Chapel Hill Chapel Hill, NC United States; 10 London School of Hygiene and Tropical Medicine London United Kingdom; 11 Washington University School of Medicine St Louis, MO United States

**Keywords:** cervical cancer prevention, clinical practice guidelines, gynecologists, implementation science, Nigeria

## Abstract

**Background:**

Cervical cancer is a common cause of death among women globally, particularly in Africa. Each year, an average of 7093 women in Nigeria die from cervical cancer. Clinical practice guidelines developed by the Society of Obstetrics and Gynecology of Nigeria (SOGON) aim to prevent cervical cancer. However, the extent of their adoption among gynecologists remains unclear.

**Objective:**

This study aimed to assess Nigerian gynecologists’ awareness, understanding, and incorporation of the SOGON clinical practice guidelines for cervical cancer prevention in their clinical practices.

**Methods:**

A convergent parallel mixed methods design was used. Quantitative data were collected via a web-based and in-person survey distributed to gynecologists attending the 57th SOGON Annual General Meeting in Kano, Nigeria (November 2023). A total of 105 gynecologists completed the survey (response rate: 80%). Key informant interviews (n=12) were conducted to provide qualitative insights. Quantitative data were analyzed using descriptive and inferential statistics, including logistic regression (*P*<.05). Thematic analysis was applied to qualitative data.

**Results:**

Among the 105 respondents (mean age 50, SD 8.3 y and mean postresidency practice 12, SD 9.4 y), 98 (93.3%) reported awareness of the SOGON guidelines, and 74 (70.5%) endorsed their importance for cervical cancer prevention. However, only 58.1% (61/105) of the respondents reported integrating the guidelines into routine clinical practice. Barriers to implementation included limited training (71/105, 67.6%), resource constraints (64/105, 60.9%), and lack of institutional support (57/105, 54.3%). Qualitative data reinforced the need for more tailored guidelines for high-risk populations and rural settings. In addition, 70.5% (74/105) of the respondents advocated for a participatory guideline review process to ensure relevance and feasibility.

**Conclusions:**

While awareness of the SOGON guidelines is high, their integration into clinical practice remains suboptimal due to systemic barriers. Strengthening training programs, improving access to resources, and enhancing institutional support are critical to increasing guideline adoption and advancing cervical cancer prevention efforts in Nigeria.

## Introduction

### Background

Globally, cervical cancer is the fourth most common cause of cancer-related death among women, with approximately half a million new cases and one-third of a million deaths annually [1]. Cervical cancer accounts for approximately 80% of global cervical cancer–related deaths [2,3]. In Africa, cervical cancer is either the leading or second leading cause of cancer-related deaths among women [4]. Despite being one of the few cancers that are 100% preventable and treatable if detected early, approximately 200,000 new cases and 80,000 deaths occur annually in sub-Saharan Africa (SSA) [4].

The risk of cervical cancer mortality is particularly high in SSA compared to other regions, such as Europe. The International Agency for Research on Cancer reports an age-standardized mortality rate of 18.9 per 100,000 women in SSA, compared to 3.4 per 100,000 women in Western Europe [5-7]. This disparity results from limited access to screening, vaccination, and treatment in SSA [8]. Many women in SSA do not have access to regular cervical cancer screening, leading to late-stage diagnosis and poor treatment outcomes [9-11]. In addition, vaccination rates against human papillomavirus (HPV), the primary cause of cervical cancer, are substantially lower in SSA compared to Europe [8,12].

In Nigeria, cervical cancer remains a substantial public health challenge due to the absence of structured screening programs, insufficient public awareness of preventive measures, and systemic health care barriers [13,14]. It is the second most prevalent malignancy among women aged between 15 and 44 years, with 13,676 new cases and 7093 deaths recorded in 2023 [15], corresponding to an age-standardized incidence rate of 18.4 per 100,000 and death rate of 3.2 per 100,000 [15-17]. Approximately 60.9 million women aged ≥15 years in Nigeria are at risk of developing cervical cancer unless proactive measures are taken [15,18]. However, these figures may underestimate the true burden due to underreporting, inadequate cancer surveillance systems, and limited data collection infrastructure [19,20]. Furthermore, cervical cancer disproportionately affects women during their economically productive years, compounding the socioeconomic impact and perpetuating cycles of poverty [21,22].

Globally, there is a growing emphasis on eliminating cervical cancer, particularly in low and middle-income countries [8,18]. The World Health Organization global strategy aims to achieve the “90–70–90” targets by 2030: vaccinating 90% of girls against the HPV, screening 70% of women by the age of 35 and 45 years, and ensuring that 90% of women diagnosed with precancerous conditions or cervical cancer receive treatment [18]. Meeting these targets in low and middle-income countries, including Nigeria, could reduce new cervical cancer cases by 97% (74 million cases) and prevent 62 million deaths by 2120 [23]. However, achieving these goals in Nigeria will require a robust health systems response, strong political commitment, and the effective implementation of evidence-based interventions [17,21].

Clinical practice guidelines are systematically developed to aid health care providers and patients in making informed decisions for specific clinical scenarios [24]. They offer evidence-based frameworks for best practices and carry legal and medical significance. However, their adoption is influenced by several factors, including awareness, perceived relevance, and systemic challenges [25-27]. In Nigeria, health care providers, particularly gynecologists, at the forefront of cervical cancer prevention, face unique challenges, such as navigating infrastructural deficits, addressing sociocultural stigmas, and managing limited resources [28]. These barriers highlight the need for targeted strategies to bridge the gap between guideline development and implementation.

To address this critical issue, the Society of Obstetrics and Gynecology of Nigeria (SOGON) has developed clinical practice guidelines aimed at cervical cancer prevention [29]. These guidelines provide evidence-based recommendations for primary and secondary prevention, including public health education [29]. Despite their potential to significantly reduce cervical cancer incidence and mortality, the extent to which these guidelines have been implemented remains poorly understood. Structural barriers, including inadequate health care infrastructure, limited provider training, and weak dissemination mechanisms, hinder their adoption. In addition, cultural perceptions, economic constraints, and low prioritization of women’s health further complicate the effective integration of these guidelines into routine clinical practice [10,11,30].

### Study Objective

This study applies implementation science principles to explore the perspectives of Nigerian gynecologists using the SOGON clinical practice guidelines. Unlike traditional approaches that prioritize patient outcomes, this study focuses on health care providers’ experiences, aiming to address the “know-do” gap—the discrepancy between established guidelines and actual clinical practices. By addressing this gap, the study provides critical insights into how the guidelines can be effectively implemented within Nigeria’s complex health care landscape, shaped by cultural, socioeconomic, and infrastructural challenges.

## Methods

### Study Design

This study used a convergent parallel mixed methods design. In this study design, quantitative and qualitative data were collected simultaneously, analyzed separately, and then merged to draw a comprehensive conclusion. The quantitative approach assessed the level of understanding, awareness, and application of the SOGON clinical practice guidelines. The qualitative component provided in-depth insights into the experiences, perceptions, and recommendations of gynecologists regarding implementing the SOGON clinical practice guidelines for preventing cervical cancer.

### Study Setting

The study was conducted during the SOGON 57th Annual General Meeting and Scientific Conference in Kano, Nigeria, from November 20 to 24, 2023, at the Bristol Palace Hotel. The theme of the conference was “the tragedy of maternal deaths in Nigeria: our collective responsibility.”

### Study Population

The study population was practicing gynecologists within Nigeria who attended the SOGON 57th Annual General Meeting and Scientific Conference, Kano.

### Eligibility Criteria

Eligible participants were qualified gynecologists with valid medical degrees and professional certifications that authorize them to practice in Nigeria. It was necessary for them to be actively engaged in practice in public or private health care settings, such as hospitals, clinics, or private practices, and to be located within the geographical boundaries of Nigeria during the study.

Gynecologists who were not actively practicing, such as those who were retired, those who were on extended leave, or those who had transitioned to nonclinical roles, were excluded to ensure that the data collected were directly relevant to the present clinical environment and standards. Individuals lacking completion of their medical degree or necessary gynecological training or practicing without a valid license were also excluded from maintaining professional standard benchmarks in the study. Health care professionals who were not specialized in gynecology, including general practitioners, nurses, and midwives, were excluded because the study specifically targeted implementing SOGON guidelines among gynecologists. Gynecologists unwilling to provide informed consent or participate in the survey or interview process were excluded. Finally, gynecologists of Nigerian origin practicing abroad were omitted, as the study focused on the adoption of SOGON guidelines within health care settings in Nigeria.

### Sample Size Determination

As the proportion of gynecologists aware of the SOGON guidelines is unknown, a conservative estimate of *p*=0.5 was used because it provides the maximum sample size—a standard CI level of 95%, which gives a *z* score of 1.96 [31]. A margin of error (*e*), the acceptable error, was set at 5% (0.05), as in many social science studies:

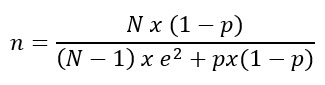

- *n*=sample size
- *N*=population size (total number of attendees at the conference)
- *p*=estimated proportion of the attribute in the population (usually set at 0.5 for maximum sample size)
- *e*=margin of error (acceptable error rate)

Therefore, a sample size of approximately 110 gynecologists was required for the study.

The qualitative component of this study involved 12 purposively selected participants for key informant interviews (KIIs). These participants were chosen based on their extensive experience or unique perspectives on the implementation of SOGON guidelines. Extensive experience was defined as having at least 10 years of clinical practice as a gynecologist or serving in leadership roles, such as heads of departments or members of guideline review committees, within their institutions or professional organizations such as SOGON. These individuals were considered to possess in-depth knowledge and expertise in the application of the guidelines across various clinical settings. Unique perspectives were sought from individuals working in underserved or rural areas, where distinctive challenges in implementing guidelines are often encountered. In addition, participants involved in educational or advocacy roles related to cervical cancer prevention were included to provide insights into the dissemination and training processes for the guidelines.

The selection process took place during the SOGON 57th Annual General Meeting, where potential participants were identified through professional networks, conference attendee lists, and peer recommendations. Invitations were extended to ensure representation from diverse geographic regions and practice settings, capturing a broad spectrum of experiences relevant to the study’s objectives.

The sample size of 12 participants was guided by the principle of thematic saturation, a widely recognized concept in qualitative research [32]. Saturation occurs when no new significant themes or insights emerge from additional data collection, indicating that the sample size sufficiently captures the diversity of perspectives needed to address the research objectives [33,34]. This point was reached during the iterative process of data collection and analysis, where the responses from the 12 interviews provided comprehensive insights into the challenges and facilitators of implementing SOGON guidelines.

### Data Collection Instruments

The data collection instruments for this study were adapted from validated scales and designed to measure the attitudes of gynecologists toward adopting the evidence-based practice (EBP), precisely the SOGON guidelines for cervical cancer screening. These adaptations were based on established scales and reviews from previous studies, such as the Evidence-Based Practice Attitude Scale [35] and the insights from the barriers of clinical guideline use among physicians [36]. The instruments were modified to assess the adoption barriers of the SOGON guidelines among Nigerian gynecologists and underwent expert review to ensure content validity.

### Quantitative Data Collection

Quantitative data were collected using a structured web-based survey divided into 3 sections. The study tool exhibited a robust internal consistency, as evidenced by an overall Cronbach α score of 0.890.

#### Section A: Sociodemographic Information

This section gathered basic demographic and professional information from the respondents, including age in years, gender, race or ethnicity, annual household income, board certification (year), number of years of postresidency practice, average number of patients seen on a typical day, and characterization of clinical practice (urban, suburban, or rural).

#### Section B: Awareness and Understanding of the SOGON Guidelines

The respondents were asked to indicate their level of agreement with statements designed to assess their awareness and understanding of the SOGON guidelines for cervical cancer screening. This session contained 8 items, and responses were measured on a Likert scale, which ranged from “strongly agree” to “strongly disagree.” The Cronbach α score for section B was 0.734.

#### Section C: Incorporation of the SOGON Guidelines Into Clinical Practice

This 8-item section evaluated the extent to which the respondents integrated the SOGON guidelines into their clinical practice. A Likert scale was used to measure agreement levels with statements reflecting attitudes toward adopting new guidelines and practices. The Cronbach α score for section C was 0.932. The final part of this 7-item section includes hypothetical scenarios to understand the potential influences on the likelihood of adopting the SOGON guidelines, which focus on the role of training, a mandate from authorities, peer influence, and personal competence in the adoption process. The Cronbach α score for this final section was 0.867.

### Qualitative Data Collection

The qualitative data collection was conducted using a semistructured interview guide to explore gynecologists’ experiences and perceptions concerning implementing the SOGON guidelines. It contextualized and enriched the quantitative findings, providing deeper insight into practitioners’ attitudes, challenges, and barriers. The interview had 2 sections: section A—challenges and barriers in implementing the guidelines and section B—recommendations for improving uptake. These were open-ended questions crafted to solicit detailed narratives encapsulating personal experiences, systemic obstacles, and practical suggestions for enhancing guideline adoption.

### Data Collection Procedure

#### Quantitative Phase

A self-administered web-based survey tool (Google Forms) was used to distribute the questionnaire. The survey tool was disseminated during the SOGON 57th Annual General Meeting and Scientific Conference to the gynecologists in attendance.

Before initiating the survey, participants were presented with an informed consent form. Only those who provided consent were allowed to proceed to complete the survey. The survey was structured to be intuitive and efficient, aiming to optimize both response rates and the quality of collected data.

To facilitate extensive participation, a comprehensive distribution approach was used. Initially, participants present at the conference were approached directly, and upon obtaining their consent, they were given access to the web-based survey link. In addition, the survey link was distributed through the conference attendees’ WhatsApp group, encouraging participation. Finally, the survey link was also shared in the SOGON WhatsApp group, specifically prompting members who had attended the conference but had not yet completed the survey to fill out the survey.

#### Qualitative Phase

The qualitative phase of this study involved 12 purposively selected KIIs conducted during the SOGON 57th Annual General Meeting and Scientific Conference. To ensure a rigorous and systematic approach to data collection, 2 trained research assistants, with prior experience in qualitative research methods and interview facilitation, conducted the interviews. Before data collection, the research assistants underwent a structured training session to align their understanding of the study objectives, ethical considerations, and interview techniques, ensuring consistency in the interview process.

Participants were contacted in advance to schedule interviews at their convenience, allowing for flexible scheduling to accommodate their professional commitments. Before each interview, participants provided written informed consent, including permission for audio recording to ensure accurate data capture. The research assistants reassured participants that all recordings would remain strictly confidential and used exclusively for research purposes. Each interview was assigned a unique identifier to maintain participant anonymity.

Interviews were conducted in a quiet and private setting within the conference venue to foster open and candid discussions. During the interviews, the research assistants took field notes, capturing nonverbal cues and contextual insights to complement the audio recordings. Immediately after each interview, postinterview reflections were documented to enhance the depth of analysis and ensure comprehensive data triangulation.

### Data Analysis

#### Quantitative Data Analysis

Data management and statistical analysis were performed using SPSS (version 27; IBM Corp). The variables were appropriately coded for analysis, with sociodemographic factors such as age, gender, and years of practice considered as independent variables and the extent of SOGON guideline integration into clinical practice as the dependent variable. Means and SDs were reported for continuous independent variables such as age and years of practice. Categorical independent variables such as gender and board certification were described using frequencies and percentages. The dependent variable was summarized using frequencies and percentages across different levels of agreement or adoption.

The questions in each section of the study were specifically designed to evaluate participants’ awareness, practices, and attitudes using a Likert scale. To assess the internal consistency of these scales, we calculated Cronbach α for each section. The results demonstrated strong reliability, with Cronbach α values of 0.846 for awareness, 0.849 for attitudes, and 0.811 for practices.

On the basis of the evidence of intercorrelation among the items, we grouped the Likert scale questions into a cohesive survey scale and calculated the mean score for these items [37]. Our analysis used the weighted mean as a benchmark: when the mean score was greater than or equal to the weighted mean, it indicated adequacy in the measured items among the participants. Conversely, a mean score below the weighted mean suggested inadequacy in the items assessed.

Logistic regression analysis was performed to identify predictors of guideline uptake, with the dependent variable representing the likelihood of guideline integration. Each independent variable’s contribution to the model was assessed, and odds ratios (ORs) were calculated to measure the strength of the association. A significance level (α) of .05 was set for all statistical tests, indicating that results with a *P* value <.05 were considered statistically significant.

#### Qualitative Data Analysis

The qualitative data analysis was conducted by 3 members of the research team (FTA, ORA, and OMO) and 2 qualitative research experts with experience in implementation science and health policy research. The qualitative data analysis was conducted using a thematic analysis approach. This approach was used to identify, analyze, and report patterns (themes) within data because it provided a flexible approach that can be applied to diverse research questions and highlight similarities and differences across a dataset.

Each audio recording from the in-depth interviews was transcribed verbatim. Transcriptions were double-checked against the recordings to ensure accuracy, capturing nonverbal cues when necessary. NVivo software (Lumivero) was used to organize codes and associate them with text segments. After the initial coding, themes were developed by collating codes and gathering all data relevant to each theme to identify patterned responses or meanings. The themes were reviewed in relation to the coded extracts and the entire dataset. They were then refined to form coherent and distinct patterns. A clear definition and name for each theme was developed, capturing the essence of each theme and what aspect of the data it represents. The final stage involved the selection of vivid and compelling extract examples, analyzing the extracted data, and relating the analysis to the research question and literature. The study detailed each theme, including how themes support, contradict, or develop existing knowledge.

Discrepant cases and nonconfirming evidence were discussed in the narrative to acknowledge the complexity and diversity of the data.

### Triangulation and Integration of Data

This study used a convergent parallel mixed methods approach, collecting quantitative and qualitative data simultaneously but analyzing them independently to maintain the integrity of each method. Integration occurred during the interpretation phase, enabling the quantitative findings to provide breadth and generalizability, while qualitative data offered depth and contextual insights. The integration process involved comparing quantitative results, such as awareness levels and predictors of guideline adoption, with qualitative themes that explored systemic barriers such as resource constraints and training deficits.

This triangulation facilitated a comprehensive understanding by identifying areas of convergence, divergence, and complementarity. Both data types consistently showed high awareness but moderate implementation, highlighting a gap between knowledge and practice. Qualitative data explained this gap, revealing resistance due to reliance on clinical judgment and contextual challenges. The combined analysis strengthened the study’s conclusions, ensuring a nuanced understanding of SOGON guideline implementation and aligning with best practices in mixed methods research to address the complexities of health systems.

### Ethical Considerations

Before the study commenced, ethics approval was sought and obtained from the National Health Research Ethics Committee (approval number NHREC/01/01/2007).

Participants were provided detailed information about the study’s purpose, procedures, potential risks, and benefits. All data collected were treated with strict confidentiality. Participants were informed that they could request a summary of the study findings after the study’s completion. No financial compensation was provided, but participants received refreshments and conference-related materials.

## Results

### Overview

A total of 120 eligible participants attended the 57th SOGON Annual General Meeting and Scientific Conference, and 105 gynecologists completed the survey, yielding a response rate of 80%. A total of 105 eligible participants were included in the study. Table 1 presents the sociodemographic characteristics of the respondents. The average age of the respondents was 50 (SD 8) years, with an average of 12 (SD 9.412) years of postresidency practice and 15 (SD 11.73) patients seen daily. The gender distribution showed that 33.3% (35/105) of the respondents were women, while 66.7% (70/105) were men.

**Table 1:** Sociodemographic characteristics of respondents (n=105).

Characteristic		Values
Age (y), mean (SD)		49.83 (8.346)
Number of years of postresidency practice, mean (SD)		11.77 (9.412)
Average number of patients seen on a typical day, mean (SD)		15.2 (11.73)
**Sex, n (%)**		
	Female	35 (33.3)
	Male	70 (66.7)
**Type of practice** **, n (%)**		
	Private—nonteaching hospital	10 (9.5)
	Private—self-employed	2 (1.9)
	Public—federal medical center	12 (11.4)
	Public—federal teaching hospital	59 (56.2)
	Public—MDA^a^	1 (1.0)
	Public—state teaching hospital	5 (4.8)
	Public—state specialist hospital	16 (15.2)
**Obstetrics and gynecology fellowship year, n (%)**		
	1981-1990	3 (2.9)
	1991-2000	11 (10.5)
	2001-2010	29 (27.6)
	2011-2020	45 (42.9)
	2021 or later	17 (16.2)
**Fellowship obtained, n (%)**		
	FMCOG^b^	17 (16.2)
	FRCOG^c^	1 (1.0)
	FWACS^d^	39 (37.1)
	2 fellowships	42 (40)
	3 fellowships	5 (4.8)
	Others	1 (1.0)
**Characterization of clinical practice, n (%)**		
	Rural	4 (3.8)
	Semiurban	20 (19)
	Urban	81 (77.1)
**Other cervical cancer prevention guidelines, n (%)**		
	RCOG^e^ only	4 (3.8)
	CSOG^f^ only	4 (3.8)
	WHO^g^ only	13 (12.4)
	ACOG	5 (4.8)
	ACOG^h^ and RCOG	8 (7.6)
	WHO and ACOG	4 (3.8)
	WHO and RCOG	25 (23.8)
	WHO, ACOG, and RCOG	42 (40)

^a^MDA: ministries, departments, and agencies.

^b^FMCOG: Fellowship of the Medical College in Obstetrics and Gynecology.

^c^FRCOG: Fellow of the Royal College of Obstetricians and Gynaecologists.

^d^FWACS: Fellowship of the West African College of Surgeons.

^e^RCOG: Royal College of Obstetricians and Gynaecologists.

^f^CSOG: Clinical and Experimental Obstetrics and Gynecology.

^g^WHO: World Health Organization.

^h^ACOG: American College of Obstetricians and Gynecologists.

Regarding the type of practice, the highest proportion of respondents, 56.2% (59/105), worked in public federal teaching hospitals, while the smallest group, 1% (1/105), worked in public ministries, departments, and agencies. This was followed by 15.2% (16/105) of the respondents who worked in public state specialist hospitals, 11.4% (12/105) in public federal medical centers, 9.5% (10/105) in private nonteaching hospitals, and 4.8% (5/105) in public state teaching hospitals, and 1.9% (2/105) of the respondents were self-employed in private practice. The respondents obtained their obstetrics and gynecology fellowship in different years, with the largest group, 26.7% (28/105), obtaining their fellowship between 2016 and 2020.

Most respondents (81/105, 77.1%) practiced in urban areas, while 19% (20/105) practiced in semiurban areas and 3.9% (4/105) in rural areas. The respondents followed different cervical cancer prevention guidelines. The most commonly followed guidelines, which were followed by 40% (42/105) of the respondents, included a combination of World Health Organization, American College of Obstetricians and Gynecologists, and Royal College of Obstetricians and Gynaecologists.

There was a diverse distribution of participants across Nigeria, with higher concentrations in major urban centers—20.9% (22/105) in Lagos and 14.3% (15/105) in Abuja. The broad geographic distribution of respondents ensured comprehensive representation, strengthening the study’s ability to capture diverse perspectives from different regions and cultural contexts across the country.

### Awareness of the SOGON Guidelines

Most of the respondents (98/105, 93.3%) were aware of the SOGON cervical cancer prevention guidelines. The respondents who were aware of the SOGON guidelines indicated their level of agreement with statements designed to assess their awareness and understanding of the SOGON guidelines for cervical cancer screening.

The analysis of participants’ awareness and understanding of the SOGON clinical practice guidelines for cervical cancer prevention demonstrates a generally high level of awareness, with a weighted mean score of 4.24 (SD 0.833) serving as the benchmark for evaluating adequacy across all assessment items.

Participants exhibited strong awareness of key strategies for prevention of cervical cancer, as evidenced by a mean score of 4.63 (SD 0.686). They also demonstrated an understanding of the need for definitive confirmation following positive screening results, with a mean score of 4.26 (SD 0.959). These findings suggest that most participants had a robust grasp of the critical elements of the guidelines.

Awareness of patient eligibility for screening with a mean score of 4.32 (SD 0.830) and the recommended frequency for screening with a mean score of 4.27 (SD 0.949) were slightly above the weighted mean, reflecting a satisfactory but not exceptional level of understanding.

Some challenges emerged in the practical application of the guidelines. Participants’ familiarity with implementing SOGON recommendations in clinical practice scored a mean of 4.06 (SD 0.858), while their ability to differentiate SOGON-advised screening methods from other screening tests scored 3.76 (SD 0.856), falling below the weighted mean. These scores highlight areas where additional training or practical tools might be required to bridge the gap between theoretical understanding and practical execution.

Knowledge about assessing patient eligibility according to the guidelines was slightly below the benchmark, with a mean score of 4.10 (SD 0.891). Although this indicates reasonable understanding, enhancing training in this area could further improve participants’ competence (Table 2).

**Table 2:** Awareness and understanding of the Society of Obstetrics and Gynecology of Nigeria (SOGON) guidelines for cervical cancer prevention.

Assessment scale	Score, mean (SD)
“I am aware that SOGON cervical cancer guidelines recommend specific strategies for cervical cancer prevention among women and girls.”	4.63 (0.686)
“I understand that women with positive screening results based on SOGON cervical cancer prevention guidelines need to undergo definitive confirmation before treatment.”	4.26 (0.959)
“I am aware that positive screening results based on SOGON prevention guidelines do not mean patients have cervical cancer.”	4.54 (0.638)
“I am aware of which patients are eligible for screening based on recommendations by SOGON cervical cancer prevention guidelines.”	4.32 (0.830)
“I am aware of the recommended frequency of screening by SOGON cervical cancer prevention guidelines for eligible women.”	4.27 (0.949)
“I am familiar with how the recommendation of the SOGON cervical cancer prevention guidelines for screening methods are applied in clinical practice.”	4.06 (0.858)
“I can differentiate between the SOGON-advised cervical cancer screening method and other cervical screening tests.”	3.76 (0.856)
“I am knowledgeable about how to assess patients eligible for screening according to SOGON guidelines.”	4.10 (0.891)
Weighted mean	4.24 (0.833)

The evaluation of professionals’ incorporation of the SOGON clinical guidelines into practice provides valuable insights into their attitudes and behaviors. The weighted mean score of 3.40 (SD 1.075) serves as a benchmark for understanding overall alignment with the guidelines, with higher scores indicating stronger agreement and adherence and lower scores highlighting potential areas of resistance or reliance on alternative approaches. Participants demonstrated a strong willingness to adopt evidence-based guidelines, as reflected by a high mean score of 4.60 (SD 0.690) for receptiveness to research-backed practices. Similarly, a mean score of 4.15 (SD 0.869) indicates a positive attitude toward adapting to new guidelines, even when these differ from established practices. These scores, well above the weighted mean, emphasize the professionals’ recognition of the importance of evidence-based approaches in clinical decision-making. While participants generally valued the guidelines, the mean score of 3.32 (SD 1.410) suggests that many professionals perceive a misalignment between guidelines and the realities of clinical practice. The results indicate a tendency to prioritize personal experience and clinical judgment over formal guidelines, with scores for statements such as “My clinical judgment often takes precedence over academic or formal guidelines” (mean 2.69, SD 1.389) and “Relying on my clinical experience is often more crucial than strictly following a guideline” (mean 2.22, SD 1.374) falling significantly below the weighted mean. These findings point to a preference for individualized decision-making, particularly in complex or unique patient cases. In addition, hesitancy to strictly follow structured guidelines is further reflected with a low mean score of 1.87 (SD 1.177). This suggests that rigid adherence may be seen as impractical or unsuitable in many clinical scenarios, emphasizing the need for guidelines that accommodate flexibility. The mean score of 4.04 (SD 1.012) for integrating new guidelines into practice is slightly above the weighted mean, indicating that while participants strive to incorporate guidelines, there is room for improvement. Therefore, the weighted mean of 3.40 (SD 1.075) represents a moderate overall level of integration, with significant variability across specific aspects of guideline adoption (Table 3).

**Table 3:** Evaluation of the extent to which you incorporate the Society of Obstetrics and Gynecology of Nigeria (SOGON) guidelines into your clinical practice.

Assessment scale	Score, mean (SD)
“I often integrate new guidelines or practices, such as the SOGON guidelines to enhance patient care”	4.04 (1.012)
“My clinical judgment, based on experience, often takes precedence over academic or formal guidelines”	2.69 (1.389)
“I am receptive to implementing guidelines or practices that are backed by research, like the SOGON guidelines”	4.60 (0.690)
“Guidelines derived from research, such as the SOGON guidelines, may not always align with clinical realities”	3.32 (1.410)
“Relying on my clinical experience is often more crucial than strictly following a guideline”	2.22 (1.374)
“I am hesitant to adhere to any structured guideline in my practice strictly”	1.87 (1.177)
“I am willing to adapt to new guidelines, even if they differ significantly from my usual practices”	4.15 (0.869)
Weighted mean	3.40 (1.075)

Table 4 shows an overall perception of adopting new guidelines and practices, as indicated by the weighted mean score of 4.45 (SD 0.782). This benchmark highlights participants’ positive attitudes toward integrating EBPs into their clinical routines. Participants showed the highest agreement with statements regarding aligning new guidelines with their clinical intuition (mean 4.69, SD 0.640) and understanding of effective practice (mean 4.70, SD 0.484). These scores, well above the weighted mean, suggest that professionals are most likely to adopt guidelines that resonate with their personal judgment and established practices. While slightly lower than the weighted mean, the score for adoption driven by supervising authority mandates (mean 4.12, SD 0.978) indicates moderate influence from hierarchical directives. However, compliance with broader regulatory requirements (mean 4.39, SD 0.860) and state or national mandates (mean 4.32, SD 0.826) scored closer to the weighted mean, reflecting professionals’ general willingness to adhere to standardized practices when required. The mean score of 4.32 (SD 0.904) for colleagues’ adoption and feedback suggests that peer practices significantly shape attitudes toward new guidelines. This highlights the importance of collaborative environments and peer networks in fostering guideline adoption. A high mean score of 4.60 (SD 0.780) indicates that participants felt well trained and competent in applying new guidelines. This score, significantly above the weighted mean, underscores the critical role of professional development and training in enhancing confidence and facilitating the integration of EBPs.

**Table 4:** Attitudes toward adopting new guidelines and practices.

Assessment scale	Score, mean (SD)
“It aligned well with your clinical intuition.”	4.69 (0.640)
“It resonated with your understanding of effective practice.”	4.70 (0.484)
“Your supervising authority mandated it.”	4.12 (0.978)
“The regulatory body required it.”	4.39 (0.860)
“It was a state or national mandate.”	4.32 (0.826)
“Many of your colleagues adopted it and provided positive feedback.”	4.32 (0.904)
“You felt sufficiently trained and competent in its application.”	4.60 (0.780)
Weighted mean	4.45 (0.782)

Table 5 shows that participants with more than 1 fellowship and a high awareness level significantly impact the adoption of SOGON clinical practice guidelines for cervical cancer prevention. The findings from the adjusted model showed that participants with more than 1 fellowship were 4 times more likely to integrate the SOGON clinical practice guidelines (OR 4.200, 95% CI 1.369-12.888). Similarly, those with a high awareness level were 9 times more likely to influence the incorporation of the SOGON clinical practice guidelines into practice (OR 9.610, 95% CI 3.146-29.357).

**Table 5:** Factors affecting the incorporation of the Society of Obstetrics and Gynecology of Nigeria (SOGON) guidelines into practice.

Variables and category		Crude OR^a^ (95% CI)	*P* value	Adjusted OR (95% CI)	*P* value
**Fellowships**					
	1	Reference	—^b^	Reference	—
	>1	3.462 (1.354-8.849)	.01^c^	4.200 (1.369-12.888)	.01^c^
**SOGON awareness**					
	No	Reference	—	Reference	—
	Yes	0.662 (0.234-1.869)	.44	0.529 (0.529-0.150)	.32
**Awareness level**					
	Low	Reference	—	Reference	—
	High	8.719 (3.104-24.488)	<.001^d^	9.610 (3.146-29.357)	<.001^c^

^a^OR: odds ratio.

^b^Not applicable.

^c^Statistically significant at *P*<.05

^d^Statistically significant at *P*<.05

The qualitative analysis revealed that while most gynecologists knew the SOGON guidelines, there were notable challenges in fully integrating them into clinical practice. Participants shared diverse experiences related to their application of the SOGON clinical practice guidelines, with key themes including practical difficulties in implementation, the need for additional training, and the impact of institutional support on adherence to the guidelines (Textbox 1). The findings provide a comprehensive understanding of the factors influencing guideline adoption and offer recommendations for improving the effectiveness of the SOGON clinical practice guidelines in clinical practice.

**Themes and subthemes.**

**Specificity and relevance**

- Applicability to patient needs
- Specificity for high-risk groups

**Accessibility**

- Availability
- Ease of understanding

**Training and capacity building**

- Experiences with training
- Additional training needs

**Institutional support and resources**

**Patient education and awareness**

- Role in guideline implementation

**Feedback and improvement**

- Feedback provision
- Participatory guideline review

**Comparison with other guidelines**

- Reference to other guidelines

**Guideline implementation successes**

- Positive experiences

### Theme 1: Specificity and Relevance

This theme explores the challenges and perceptions of gynecologists regarding the implementation of the SOGON clinical practice guidelines, focusing on their applicability to the unique needs of their patients and specificity for high-risk groups. The responses reveal diverse perspectives, underscoring the importance of aligning guidelines with local and specific patient contexts.

#### Subtheme 1.1: Applicability to Patient Needs

Gynecologists shared their experiences on how well the SOGON guidelines meet the diverse needs of their patients, particularly those in underserved areas. The responses highlight a perceived gap in the guidelines’ relevance for these populations and suggest a need for tailored approaches.

While the guidelines provide a broad framework, there is a consensus among respondents that they lack flexibility for rural and socioeconomically disadvantaged populations, necessitating frequent updates and training for practitioners:

The SOGON guidelines are comprehensive but not always a perfect fit for the unique needs of my patients in rural areas. There is a lot that needs to be done for the rural communities. I have colleagues who have not been able to do much in their communities; there is a need for regular training and updates on the SOGON guidelines. Some challenges are also socioeconomic conditions, for which I believe there is a need for more tailored approaches than the guidelines provided.

Participant 009

#### Subtheme 1.2: Specificity for High-Risk Groups

Gynecologists emphasized the importance of adapting the guidelines for high-risk groups, such as women with a family history of cervical cancer or those living with HIV.

Participants identified a critical need for more detailed and tailored guidance when managing high-risk populations. They suggested that additional specificity would enhance clinical decision-making and improve patient outcomes:

I appreciate the guidelines, but there is a need for more specificity, especially when dealing with high-risk groups. We need to do more.

Participant 001

For certain cases, like high-risk patients, having more detailed guidance would enhance our practices.

Participant 002

### Theme 2: Accessibility

Successful implementation of clinical practice guidelines depends on their accessibility and ease of comprehension for health care providers. This theme addresses gynecologists’ experiences with accessing and understanding the SOGON guidelines.

#### Subtheme 2.1: Availability

Participants reflected on the availability of the guidelines, acknowledging it as a positive first step but expressing concerns about awareness among practitioners.

While availability is essential, there is a clear indication that more needs to be done to raise awareness, particularly among new residents and rural health care workers, to ensure widespread adoption:

The guidelines are available, which is a good starting point for adherence across practitioners.

Participant 004

Accessing the guidelines are crucial; it is the first step in ensuring everyone can follow the best practices. However, I will tell you that some of us are unaware that such guidelines exist, especially for new residents. Then I ask myself, what are we doing then? I believe there is a need to do more awareness even during a Conference like this, though the guidelines are available.

Participant 002

#### Subtheme 2.2: Ease of Understanding

Gynecologists discussed the need for user-friendly formats to facilitate the quick and easy application of guidelines in their busy clinical settings.

The data suggest that while the guidelines are present, their practical utility is limited by their complexity. The respondents advocated for simplified formats, such as pictorial aids and ongoing training, to enhance comprehension and adherence:

The guidelines are there, but they are not always easy to digest quickly during busy days. I suggest we have them in pictorial formats for ease of understanding. Also, regular training and workshops will make it easier to understand.

Participant 001

To enhance adherence, we need more user-friendly formats, especially during hectic clinic hours. I am sure you understand what I am talking about. I see more than 10 patients a day aside from administrative positions and other things I need to do. A conference like this, which is some days off work, could be a good platform to revisit the guidelines, if possible not only the SOGON but other guidelines.

Participant 005

### Theme 3: Training and Capacity Building

Training is crucial for empowering gynecologists to effectively implement clinical practice guidelines. This theme reflects their experiences and needs regarding training on the SOGON guidelines.

#### Theme 3.1: Experiences With Training

The respondents acknowledged the benefits of past training but emphasized the need for continuous, focused sessions on the guidelines.

There is a recognition that training efforts have been beneficial but insufficient. The participants expressed the need for more structured and regular training opportunities to stay updated with best practices:

I’ve had some training, but there is always room for more focused sessions on SOGON guidelines. This is very important to get this right.

Participant 009

Training has been helpful, but it needs to be ongoing to keep up with updates and best practices. Why not have a pre-conference training for this ongoing conference? This is very important.

Participant 007

#### Subtheme 3.2: Additional Training Needs

The respondents highlighted specific areas where additional training would enhance their ability to implement the guidelines effectively.

The feedback showed that participants are eager for deeper, targeted training, particularly on complex aspects of the guidelines that require further explanation:

Identifying specific areas where additional training is needed can significantly enhance our ability to implement guidelines effectively.

Participant 009

There is a need for more support, especially in areas where the guidelines require a deeper understanding. There is a need for additional and regular training.

Participant 011

### Theme 4: Institutional Support and Resources

Gynecologists operate within health care systems that either facilitate or hinder guideline implementation. This theme examines the influence of institutional support on adherence to clinical practice guidelines.

The data reveal that institutional backing is a significant determinant of successful implementation. The gynecologists expressed that without adequate support, adherence remains challenging, thus affecting patient care quality:

Without institutional support, adherence to guidelines becomes challenging, directly affecting patient care. In my workplace, it is difficult to convince my boss to adopt the guidelines.

Participant 012

Instances where our institution provided crucial support stand out as successful implementations of the guidelines. I can tell you that support is key, and I am a witness in my formal workplace.

Participant 011

### Theme 5: Patient Education and Awareness (Subtheme 5.1: Role in Guideline Implementation)

The gynecologists recognize that cervical cancer prevention extends beyond clinical practice, requiring active patient education to ensure guideline adherence.

The respondents described how educating patients plays a critical role in implementing guidelines successfully.

The feedback underscores the need for a collaborative approach between practitioners and patients. Educating patients not only helps in adherence but also empowers them to take proactive steps for their health:

Educating patients is integral to successful implementation; it is a partnership between practitioners and those we serve.

Participant 009

Our approach to patient education significantly influences how well the guidelines are understood and followed.

Participant 008

### Theme 6: Feedback and Improvement

This theme discusses the importance of structured feedback mechanisms in improving and updating the SOGON guidelines.

#### Theme 6.1: Feedback Provision

The gynecologists emphasized the value of providing feedback as a means to refine the guidelines based on practical experiences.

The responses indicate a strong desire for a more systematic process where practitioners can share their experiences and insights to contribute to continuous improvement.

Some respondents stated the following:

Providing feedback is essential; it is a way to contribute to improving the guidelines.

Participant 012

There is a need for a structured process where practitioners can easily provide feedback on their experiences.

Participant 009

#### Theme 6.2: Participatory Guideline Review

Furthermore, 70% (74/105) of the participants advocated for a participatory approach in the guideline review process, ensuring their experiences inform updates.

The gynecologists’ input suggests that a participatory model would enhance trust and ownership among practitioners, leading to more effective guideline implementation.

Some of the respondents said the following:

Making the guideline review more participatory ensures that practitioners’ insights are considered in updates.

Participant 008

We want to be part of the process, contributing our experiences to refine the guidelines effectively.

Participant 007

It is not that I do not trust the SOGON guidelines; just to inform you, it was not even mentioned during my years of residence. However, being in a scientific profession, I think we want whatever decision or guideline taken to be backed up by “We are doing this because it is safer. It is safest for the patients, and this is the evidence,” rather than “We are doing this,” without explaining because you might think: Oh, you are doing this. After all, SOGON says it works.

Participant 009

### Theme 7: Comparison With Other Guidelines (Theme 7.1: Reference to Other Guidelines)

This theme highlights how gynecologists compare the SOGON guidelines with other standards to provide holistic care.

The participants discussed their practice of consulting various guidelines to ensure comprehensive patient care.

Comparing guidelines appears to be a common strategy among gynecologists to incorporate best practices from multiple sources, suggesting that the SOGON guidelines could benefit from integrating insights from other frameworks.

The gynecologists pointed out the following:

Sometimes, we refer to other guidelines; it is essential to consider a holistic approach to patient care.

Participant 002

Comparing guidelines helps us understand where SOGON stands and how to integrate the best practices.

Participant 001

### Theme 8: Guideline Implementation Successes (Theme 8.1: Positive Experiences)

This theme focuses on successful instances of guideline implementation and the factors that contributed to these outcomes.

The gynecologists shared successful strategies they have used, highlighting the importance of collaboration and adaptability.

The success stories showed that with the right support and approach, effective implementation is achievable. Such examples serve as learning opportunities for other practitioners:

Sharing our successes allows us to learn from each other and replicate effective strategies.

Participant 003

Factors contributing to successful implementations are valuable lessons for the entire community.

Participant 006

## Discussion

### Overview

This study was motivated by the introduction of SOGON clinical practice guidelines aimed at enhancing cervical cancer prevention in Nigeria. Despite the availability of these guidelines, data on their adoption by gynecologists remain limited. Understanding the awareness, implementation, and barriers to guideline adherence is critical for improving cervical cancer prevention practices.

### Principal Findings

This study used a convergent parallel mixed methods design to evaluate the awareness, understanding, and application of the SOGON guidelines among Nigerian gynecologists. Quantitative data from surveys quantified awareness levels (98/105, 93.3%) and practice distribution (81/105, 77.1% urban), while qualitative data from KIIs provided contextual insights into barriers, such as limited training and institutional support. Integration occurred at the interpretation stage, where quantitative findings, such as the significant association between awareness and guideline adoption (OR 9.610, 95% CI 3.146-29.357), were enriched by qualitative narratives emphasizing professional development needs and rural-urban disparities. This approach revealed systemic and contextual challenges, including resistance to strict adherence due to overreliance on clinical judgment. These findings align with existing literature, highlighting the importance of combining quantitative and qualitative methods to address the complexities of clinical guideline implementation [38,39].

The high levels of awareness and understanding demonstrated by gynecologists align with EBPs aimed at optimizing patient care [18,40]. Participants knew the need for confirmatory diagnostics following positive screening results, minimizing unnecessary interventions and patient anxiety [29,41]. However, the moderate alignment of clinical practice with the guidelines reflects ongoing challenges in bridging the gap between knowledge and implementation. Global studies similarly highlight that gaps in practical application are often due to insufficient training and limited resources, underscoring the critical role of continuing education in strengthening guideline adoption [38,42,43].

Integrating the SOGON guidelines into clinical practice revealed moderate alignment with the recommendations. The positive perception of adopting new guidelines indicates that health care professionals are generally open to embracing EBPs [42]. This finding aligns with the findings from the study by Lehane et al [39], who highlighted that educational programs and associated curricula are pivotal in shaping health care professionals’ knowledge, skills, and attitudes, influencing the quality of care delivered. Such programs provide the foundational support necessary to bridge the gap between guidelines and their practical application [39,44]. Despite this positive outlook, the study identified a reliance on clinical judgment over formal guidelines and a hesitancy to adhere strictly to structured recommendations. This underscores the complex interplay between clinical expertise and guideline adherence in real-world settings, where health care providers often navigate patient-specific needs and systemic constraints. Achieving a balance between respecting clinical judgment and prioritizing evidence-based guidelines is critical to optimizing patient outcomes [44,45]. A systematic review has categorized 3 critical strategies for enhancing skills in EBPs: multifaceted educational approaches that incorporate mentoring and tutoring, single educational strategies, and multifaceted approaches using the 5 fundamental steps of EBPs. These strategies have demonstrated effectiveness in improving health care professionals’ ability to integrate evidence-based guidelines into routine practice, emphasizing the importance of targeted training initiatives to strengthen guideline adoption [39,46].

The study findings highlight that Nigerian gynecologists positively embrace new guidelines, even when these differ significantly from their established practices. This openness reflects a commitment to continuous learning and improving patient care, aligning with fostering a culture of lifelong learning and professional development within health care systems [44,47]. This perspective is consistent with evidence from prior studies, which emphasize that adopting new guidelines impacts far beyond their immediate content, shaping broader attitudes and behaviors in clinical practice [26,27,39,48].

The results provide valuable insights into the factors influencing gynecologists’ perceptions and adoption of new guidelines. The participants demonstrated strong agreement regarding the importance and efficacy of adopting guidelines, particularly when these align with their clinical intuition and understanding of effective practice. This finding underscores the role of clinical expertise and professional judgment, which health care providers often rely upon to navigate complex and diverse clinical scenarios. Global studies have observed similar trends, where clinical decision-making is frequently informed by a combination of evidence-based guidelines and contextual knowledge of patient needs [11,21,49]. The study also highlights the importance of involving clinicians in the guideline development process to ensure that recommendations resonate with their practical realities. Guidelines tailored to incorporate clinicians’ perspectives are more likely to be adopted as they address theoretical best practices and the day-to-day challenges faced in diverse clinical settings. This finding aligns with the literature emphasizing the need for participatory approaches in guideline development to enhance relevance and feasibility [38]. Interestingly, the study revealed that while regulatory requirements and mandates promote adherence, they are not the primary drivers of guideline adoption. The relatively lower mean score associated with the influence of supervisory mandates indicates that clinicians are more motivated by the intrinsic value of guidelines and their alignment with clinical judgment rather than top-down enforcement. Nonetheless, the participants demonstrated high compliance with requirements from regulatory bodies and national policies, underscoring the importance of oversight mechanisms in ensuring standardized practices and maintaining quality of care. Similar findings in the literature highlight the complementary role of regulatory frameworks in reinforcing the integration of EBPs [50-53].

The study highlights the significant role of peer support and professional networks in shaping participants’ perceptions and adoption of new guidelines. The influence of colleagues’ adoption and feedback was key in promoting guideline adherence and knowledge sharing among Nigerian gynecologists [48]. Collaboration and peer learning opportunities facilitate the exchange of best practices, fostering a culture of continuous improvement within health care organizations. These findings are consistent with prior studies, demonstrating that strong professional networks and peer endorsements enhance the credibility and acceptability of EBPs [38,54]. Participants’ confidence and self-efficacy in applying the SOGON guidelines were closely linked to their perception of being well trained and competent. This underscores the importance of ongoing training and professional development initiatives in ensuring health care professionals’ proficiency in delivering evidence-based care. Research consistently shows that well-designed training programs improve clinicians’ confidence and ability to implement clinical guidelines, particularly when they incorporate hands-on practice and tailored content to address specific needs [9,55].

The study also provides insights into factors influencing the adoption of the SOGON guidelines. A notable finding was the significant impact of educational background and awareness levels on guideline integration. Gynecologists with more than 1 fellowship were significantly more likely to adopt the guidelines than those with only 1 fellowship, emphasizing the value of advanced training and specialization in enhancing knowledge and skills. This finding aligns with global evidence that advanced professional development improves clinicians’ ability to interpret and apply EBPs in diverse clinical settings [44,45]. Furthermore, the study revealed that gynecologists with high awareness of the SOGON guidelines were 9 times more likely to use them in their practice than those with lower awareness levels. This highlights the critical role of knowledge dissemination and awareness campaigns in promoting guideline adherence and standardizing clinical practices. Prior studies have similarly demonstrated that targeted educational initiatives, such as workshops and seminars, significantly improve guideline uptake by addressing knowledge gaps and enhancing familiarity with the guidelines [47,56]. These findings have important implications for clinical practice and professional development strategies in Nigerian health care. To facilitate the widespread adoption of EBPs, health care organizations and policy makers should prioritize educational programs and awareness campaigns focused on improving health care professionals’ knowledge of the SOGON guidelines. This could include integrating guideline training into residency programs, hosting regular workshops, and developing accessible learning materials tailored to clinicians’ needs. In addition, encouraging continued professional development and advanced training opportunities will further enhance health care professionals’ capacity to effectively integrate guidelines into their practice, ultimately improving patient outcomes [47,57].

This study provides valuable insights into gynecologists’ experiences and perceptions regarding implementing the SOGON guidelines for cervical cancer prevention. While participants acknowledged the comprehensiveness of the guidelines, many highlighted the need for greater tailoring to address the unique needs of rural and high-risk patient populations. These findings align with existing literature emphasizing the importance of context-specific adaptations to clinical guidelines to ensure their relevance and practicality in diverse health care settings [25,38].

Accessibility emerged as a critical factor influencing adherence to guidelines. Although participants recognized that the guidelines were available, concerns about their awareness, comprehensibility, and ease of use were frequently raised, particularly during busy clinical hours. Participants recommended improving accessibility through user-friendly formats, such as simplified language, visual aids, and digital tools. These suggestions are consistent with prior research, demonstrating that clear, visually engaging materials and practical demonstrations significantly improve guideline uptake among health care providers [39,58]. In addition, regular training sessions were emphasized as essential for promoting guideline adherence and enhancing gynecologists’ knowledge and skills in cervical cancer prevention. Continuous education opportunities keep health care providers updated on the latest EBPs and foster a culture of lifelong learning and professional growth [52,59-61].

Participants also emphasized the role of institutional support in facilitating guideline adherence. Successful implementation of the SOGON guidelines was often associated with robust institutional backing, including resource allocation, leadership support, and infrastructure enhancements. This finding underscores the critical role of organizational commitment in promoting EBPs. Supporting evidence from global studies highlights that health care institutions prioritizing guideline integration through dedicated resources and consistent leadership achieve higher compliance rates among health care providers [52,53]. For instance, a study in Germany demonstrated that institutions fostering an environment of quality improvement, supported by strong leadership and infrastructure, significantly enhanced guideline adherence [48,62].

Hung et al [52] investigated the role of organizational culture in influencing guideline adherence within primary care settings. Their findings demonstrated that institutions fostering a culture of continual quality improvement and consistent support and training for health care providers were significantly more effective in implementing clinical guidelines [52]. These insights reinforce the critical role of organizational commitment and institutional support in facilitating guideline integration. The participants in this study similarly highlighted the importance of institutional backing, emphasizing that robust infrastructure, leadership support, and ongoing training were essential for fostering adherence to the SOGON guidelines.

Patient education emerged as another key factor in the effective implementation of guidelines. Participants emphasized the necessity of health care practitioners educating patients and adopting a collaborative approach to care. They also advocated feedback mechanisms, calling for participatory guideline review processes to ensure continual improvement. These findings align with existing research, which shows that health care providers who actively involve patients in their care, offer clear and comprehensible information about treatment options, and encourage shared decision-making are more likely to adhere to clinical guidelines [63]. Studies have further demonstrated that health care institutions incorporating feedback from health care providers and patients into guideline review processes achieve better adherence and improved quality of care delivery [64,65]. This underscores the importance of stakeholder involvement in guideline development and refinement, ensuring that the guidelines remain relevant, practical, and patient centered.

The participants emphasized the importance of practitioners’ experiences and insights in effectively refining and updating the guidelines. The study findings show that the participants acknowledged the utility of comparing guidelines to understand best practices and ensure a holistic approach to patient care. Reference to other guidelines was perceived as valuable in guiding decision-making and practice. These findings echo evidence from prior studies that demonstrate how clinical guidelines can enhance the quality of clinical decisions by providing explicit recommendations, supporting clinicians who are uncertain about how to proceed, and addressing inconsistencies in care delivery. Guidelines grounded in rigorous scientific evidence identify beneficial interventions and highlight those lacking strong support, warning clinicians against ineffective, harmful, or wasteful practices [25,39,48]. Such evidence-based guidelines foster critical appraisal among clinicians and strengthen the consistency and appropriateness of care.

### Recommendations

On the basis of the study findings, several recommendations can enhance the implementation of SOGON guidelines and improve cervical cancer prevention among Nigerian gynecologists. Comprehensive training programs should be developed to address gynecologists’ specific needs, including guideline updates, practical implementation strategies, and clinical decision-making processes. Regular workshops, seminars, and webinars should support ongoing professional development and ensure health care providers remain updated on EBCs. Integrating SOGON guideline training into residency programs for obstetrics and gynecology will expose trainees to EBCs early, fostering a solid foundation for guideline adherence. In addition, advocacy for institutional, regional, and national policy support is critical. Efforts should focus on establishing policies that mandate guideline adherence, allocating resources for training, and incentivizing compliance to ensure widespread and effective adoption.

### Strengths and Limitations

The study’s strengths include its mixed methods approach, which combined broad quantitative data with detailed qualitative insights for a comprehensive understanding. This study is among the first to examine the adoption of cervical cancer prevention guidelines among Nigerian gynecologists. In addition, including participants from the 57th SOGON Annual General Meeting provided access to a diverse and representative group of experts in Nigeria. Despite these strengths, the study has several limitations when interpreting the findings. First, the focus on gynecologists excludes other health care professionals involved in cervical cancer prevention, such as nurses or primary care providers, limiting the generalizability of the findings. Second, while participants were selected from diverse regions, the sample size was small and may not fully represent the experiences of gynecologists in rural or underserved areas, where guideline implementation challenges may differ significantly. Third, the reliance on self-reported data introduces potential bias, as participants may overestimate adherence or underreport barriers to align with perceived expectations. Fourth, the study’s cross-sectional design prevents establishing causal relationships between factors influencing guideline adoption and their outcomes. Finally, the resource and institutional challenges reported by respondents may not fully reflect the experiences of gynecologists across different regions, given the variability in health care infrastructure and support systems within Nigeria.

### Conclusions

This study sheds light on Nigerian gynecologists’ perceptions of the SOGON guidelines for cervical cancer prevention, revealing overall positive awareness and understanding. The findings underscore the importance of guideline adherence in promoting EBPs and ensuring optimal patient care. While areas for improvement exist, such as enhancing the use of the guidelines in clinical practice, the willingness of health care professionals to adopt new guidelines indicates a commitment to continuous improvement in patient care delivery. Recommendations stemming from this study emphasize the need for comprehensive training programs tailored to gynecologists, ongoing professional development initiatives, and policy support to prioritize guideline integration.

## Abbreviations

EBP: evidence-based practice
HPV: human papillomavirus
KII: key informant interview
OR: odds ratio
SOGON: Society of Obstetrics and Gynecology of Nigeria
SSA: sub-Saharan Africa

## Data Availability

The data supporting the findings of this study are available from the corresponding author upon reasonable request. All data generated or analyzed during the study are included in this published paper and its supplementary materials.
